# Direct Measurements of Abdominal Visceral Fat and Cognitive Impairment in Late Life: Findings From an Autopsy Study

**DOI:** 10.3389/fnagi.2019.00109

**Published:** 2019-05-07

**Authors:** Aline Nishizawa, Anderson Cuelho, Daniela S. de Farias-Itao, Fernanda M. Campos, Renata E. P. Leite, Renata E. L. Ferretti-Rebustini, Lea T. Grinberg, Ricardo Nitrini, Wilson Jacob-Filho, Carlos A. Pasqualucci, Claudia K. Suemoto

**Affiliations:** ^1^Department of Pathology, University of São Paulo Medical School, São Paulo, Brazil; ^2^Department of Biomedicine, Federal University of ABC, São Paulo, Brazil; ^3^Experiment Pathophysiology Program, University of São Paulo Medical School, São Paulo, Brazil; ^4^Division of Geriatrics, University of São Paulo Medical School, São Paulo, Brazil; ^5^Department of Medical Surgical Nursing, University of São Paulo School of Nursing, São Paulo, Brazil; ^6^Department of Neurology, Memory and Aging Center, University of California, San Francisco, San Francisco, CA, United States; ^7^Department of Neurology, University of São Paulo Medical School, São Paulo, Brazil

**Keywords:** aging, autopsy, obesity, dementia, abdominal fat

## Abstract

**Background**: The relationship between cognitive impairment and abdominal visceral is controversial. Moreover, all studies so far used imaging studies to evaluate visceral fat and this association has not been described yet using autopsy material, which allows the direct quantification of abdominal fat. We aimed to investigate the association between direct measurements of abdominal visceral fat and cognitive impairment in an autopsy study.

**Methods**: In this cross-sectional study, we collected information on sociodemographics, cardiovascular risk factors, and cognitive status from subjects aged 50 or older at time of death in a general autopsy service in Brazil. Abdominal visceral fat was obtained *in natura* by the dissection of perirenal, mesenteric, omental, and mesocolon fat. The associations of total abdominal visceral fat with cognitive impairment [clinical dementia rating (CDR) score ≥0.5] and CDR-sum of boxes (CDR-SB) were evaluated using logistic regression and negative binomial regression models, respectively. All analyses were adjusted for height, age, sex, education, hypertension, diabetes mellitus, stroke, smoking, alcohol use, and physical inactivity. In addition, we compared the discrimination of visceral fat, body mass index (BMI), and waist circumference (WC) measurements in predicting cognitive impairment.

**Results**: We evaluated 234 participants (mean age = 71.2 ± 12.9 years old, 59% male). Abdominal visceral fat was inversely associated with cognitive impairment (OR = 0.46, CI = 0.30; 0.70, *p* < 0.0001) and with CDR-SB scores (*β* = −0.85, 95% CI = −1.28; −0.43, *p* < 0.0001). When we compared the area under the ROC curve (AUC), visceral fat (AUC = 0.754), BMI (AUC = 0.729), and WC (AUC = 0.720) showed similar discrimination in predicting cognitive impairment (*p* = 0.38).

**Conclusion**: In an autopsy study, larger amount of directly measured abdominal visceral fat was associated with lower odds of cognitive impairment in older adults.

## Introduction

Dementia is a common disease among the older population, affecting around 50 million people worldwide with projections indicating that dementia will affect 152 million by 2050 (Alzheimer’s Disease International, 2010; WHO, 2017). It is also a main cause of disability and dependency among older people (Alzheimer’s Disease International, 2010; WHO, 2017). Similarly, obesity prevalence had almost tripled in the last 40 years, leading to an increased risk for cardiovascular diseases (Bastien et al., 2014; Ebbert et al., 2014; Mandviwala et al., 2016; WHO, 2018). Results on the relationship between obesity and dementia are conflicting. Being overweight or obese in midlife was associated with higher risk of dementia in later life (Whitmer et al., 2005; Hassing et al., 2009; Pedditizi et al., 2016). However, the association of late-life obesity and dementia in unclear with studies showing a reverse (Buchman et al., 2005; Dahl et al., 2008; West and Haan, 2009; Cronk et al., 2010; Power et al., 2011; Pedditizi et al., 2016), a positive (Gustafson et al., 2003), and also no association (Luchsinger et al., 2007) between obesity and dementia in late-life.

Although most of the previous studies have evaluated adiposity using body mass index (BMI) as an assessment of obesity, this anthropometric measurement may not the best marker of adiposity because it cannot distinguish between fat and lean mass, nor between visceral abdominal fat and subcutaneous abdominal fat (Cereda et al., 2007). The validity of BMI as a measure of adiposity is especially problematic in older adults, who experience changes in body composition, such as decrease in muscle mass and bone mineralization, and increase in body fat (Zamboni et al., 1997; Noel and Reddy, 2005). Other measurements of adiposity, such as waist circumference (WC), waist-to-hip ratio (WHR), and abdominal visceral fat may be more appropriate in this age group (Hassing et al., 2009). In mid-life, some anthropometric measurements such as skinfold thickness (Whitmer et al., 2005) and sagittal abdominal diameter (Whitmer et al., 2008) were associated with higher risk of dementia in late life. On the other hand, prior studies have shown a positive (West and Haan, 2009) or no association of WC and cognitive impairment with dementia in late-life (Luchsinger et al., 2007; Yoon et al., 2012). Inverse associations of WHR with dementia and hippocampal volume were reported (Power et al., 2011), as well as a positive association with white matter hyperintensities (Jagust et al., 2005).

Few studies investigated the association between abdominal visceral fat and cognitive impairment. These studies used imaging methods to evaluate the amount of visceral adipose tissue (Kamogawa et al., 2010; Yoon et al., 2012; Spauwen et al., 2017). Prior studies showed no association between abdominal visceral fat area (Kamogawa et al., 2010; Spauwen et al., 2017) with cognitive impairment, while another found a positive association between visceral adipose tissue and poor cognitive performance, but only in participants younger than 70 years (Yoon et al., 2012). Autopsy studies can generate more reliable data due to the possibility of direct measurements of visceral fat surrounding different abdominal organs, as well as the total amount of visceral adiposity (van der Kooy and Seidell, 1993). However, to our knowledge, the association between cognitive impairment and abdominal visceral fat has not been investigated using autopsy material. Therefore, we aimed to evaluate this association in a large population-based autopsy study.

## Materials and Methods

### Participants

This cross-sectional study was conducted at the São Paulo Autopsy Service (SPAS) from University of São Paulo. Data were collected from October 2011 to April 2014. The SPAS performs autopsy in individuals who died from non-traumatic causes of death with unclear diagnosis during life in São Paulo, Brazil (Grinberg et al., 2007). This study was approved by the institutional review board, and the informant who agreed to participate in the study signed a written informed consent.

We included individuals aged 50 years or older at time of death, who had an informant with at least weekly contact with the deceased in the last 6 months prior to death. Exclusion criteria were inability to obtain reliable data from the informant, post-mortem interval >24 h, weight loss of 10% or more in the last 6 months prior to death, signs of autolysis according to Crossley criteria (Crossley, 1974), and retained material by the pathologist.

### Sociodemographic and Clinical Data

Information about sociodemographic data (age, sex, race, marital status, and education), frequency of contact of the informant with the deceased, and cardiovascular risk factors (current smoking and alcohol use, physical inactivity, hypertension, diabetes mellitus, and stroke) were obtained with the informant using a semi-structured interview. The cause of death and the post-mortem interval were collected from the autopsy report. Weight and height were measured with the deceased without clothes in the supine position. BMI was calculated by dividing the weight in kilogram by the squared height in meters. WC was measured with an inelastic tape in the region of the umbilicus (Nishizawa et al., 2016).

### Cognitive Assessment

We evaluated the cognitive impairment using the Clinical Dementia Rating (CDR) scale (Hughes et al., 1982; Morris et al., 1997), which contains questions regarding six areas involved in cognition (memory, orientation, judgment and problem solving, community affairs, home and hobbies, and personal care). We used only the informant part of the CDR scale due to study design. Individuals were classified into five groups: no impairment (CDR = 0); questionable dementia (CDR = 0.5); mild dementia (CDR = 1); moderate dementia (CDR = 2); and severe dementia (CDR = 3). Individuals with CDR = 0 were considered with normal cognition, and those with CDR ≥ 0.5 were considered with cognitive impairment (Suemoto et al., 2017). We also used the sum of the boxes for each domain for the CDR sum of boxes (CDR-SB) score, which ranged from 0 to 18 (O’Bryant et al., 2008).

### Evaluation of Abdominal Visceral Fat

The abdominal visceral fat was obtained by the *in natura* dissection of perirenal, mesenteric, omental, and mesocolon fat, and weighed using a calibrated electronic scale (Toledo Brazil^®^ model 3,400/05). The weight values were expressed in grams (g). To avoid measurement error, we were careful to calibrate the scale before each use. Subsequently, the values of all fat deposits were summed to obtain a measure of the total abdominal visceral fat and expressed in kilograms (kg; Nishizawa et al., 2016).

### Assessment of Other Adiposity Measurements

We measured the deceased’s weight in kg using a calibrated electronic scale, and the height in centimeters (cm) using a stadiometer. Both measurements were performed with the individual in supine position and without any clothes. Then, we calculated the BMI in kg/m^2^. We measured the WC in the umbilicus region. The abdominal subcutaneous tissue thickness (ASTT) was measured at the abdominal midline, 4 cm above the umbilicus. WC and ASTT were in cm, using an inelastic tape (Nishizawa et al., 2016).

### Statistical Analysis

A sample size of 200 participants was estimated using a power of 80%, an alpha level of 5%, and a medium effect size of 0.3 for the correlation between abdominal visceral fat and cognitive impairment in two-tailed tests (Cohen, 1992). We used mean and standard deviation (SD) for quantitative variables, or absolute and relative frequency for categorical variables to describe the sample characteristics. Sociodemographic and cardiovascular risk factors were compared among individuals with and without cognitive impairment, using unpaired *t*-test for continuous variables, and chi-square test for categorical ones.

The dependent variables were cognitive impairment evaluated by a binary variable (CDR categorized into 0 and ≥0.5) and by a continuous variable (CDR-SB); and the independent variable was the amount of abdominal visceral fat (continuous variable). As a sensitivity analysis, we also investigated the association between visceral fat and dementia (CDR ≥1), excluding those with CDR = 0.5. To evaluate the association between abdominal visceral fat and cognitive impairment, we used logistic regression. The association between abdominal visceral fat and CDR-SB was evaluated using negative binomial regression. Both analyses were adjusted for height, age, sex, education, hypertension, diabetes mellitus, stroke, smoking, alcohol use, and physical inactivity. Based on the fact that the association between obesity and CDR-SB could be different according to age groups (Yoon et al., 2012), we also included an interaction term between age and visceral fat. Finally, we calculated measures of abdominal visceral fat accuracy and compared the discrimination of visceral fat, BMI, WC, and ASST measurements in predicting cognitive impairment using the area under the receiver operating characteristic curves (AUC). We then compared the AUC using the nonparametric methods described by DeLong et al. (1988). The alpha level was set at 0.05 in two-tailed tests. We used Stata 12 (StataCorp., College Station, TX, USA) to perform the statistical analyses.

## Results

**Figure 1:**
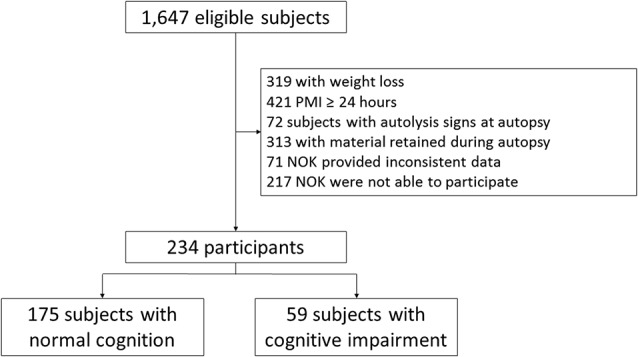
Flowchart of study participants. PMI, post mortem interval; NOK, next of kin.

During the study period, 1,647 subjects were eligible to participate in this study. Two-hundred and thirty-four subjects met the eligibility criteria for this study (Figure 1). Included and excluded subjects had similar age (Included: 71.16 ± 12.98; excluded: 70.02 ± 12.20; *p* = 0.19), and sex distribution (Included: 58.5% men; excluded: 58.2% men; *p* = 0.93). Thus, the study sample had similar demographic characteristics to the source population. Compared to participants with normal cognition, the 59 participants (25%) with cognitive impairment were older and mostly female. In addition, participants with cognitive impairment had more stroke, were more physically inactive, had lower values of BMI, and lower amount of abdominal visceral fat (Table 1).

**Table 1:** Sample characteristics according to cognitive impairment status (*n* = 234).

Variables	Total	CDR = 0 (*n* = 175)	CDR ≥ 0.5 (*n* = 59)	*p*
Age (years), mean (SD)*	71.2 (12.9)	68.3 (11.8)	79 (12.8)	<0.0001
Male, *n* (%)^†^	137 (58.5)	112 (64.0)	25 (42.4)	0.004
White, *n* (%)^†^	147 (62.8)	110 (62.9)	37 (62.7)	0.98
Married, *n* (%)^†^	114 (48.7)	94 (53.7)	20 (33.9)	0.01
Education (years), mean (SD)*	5.1 (3.8)	5.5 (3.9)	3.7 (3.1)	0.002
Daily contact of the informant with the deceased, *n* (%)^†^	194 (82.9)	145 (82.9)	49 (83.0)	0.97
Cardiovascular cause of death, *n* (%)^†^	173 (73.9)	141 (80.6)	32 (54.2)	<0.0001
Hypertension, *n* (%)^†^	172 (76.8)	128 (77.6)	44 (74.6)	0.64
Diabetes mellitus, *n* (%)^†^	72 (32.1)	54 (32.7)	18 (30.5)	0.76
Stroke, *n* (%)^†^	35 (15.6)	17 (10.2)	18 (30.5)	<0.0001
Current smoking, *n* (%)^†^	65 (28.0)	58 (33.3)	7 (11.9)	0.01
Current alcohol use, *n* (%)^†^	74 (31.9)	67 (38.5)	7 (12.1)	0.001
Physical inactivity, *n* (%)^†^	158 (67.5)	106 (60.6)	52 (88.1)	<0.0001
BMI (kg/m^2^), mean (SD)*	23.8 (5.9)	25.0 (5.4)	20.2 (5.8)	<0.0001
WC (cm), mean (SD)*	89.8 (15.5)	93.0 (14.0)	80.3 (16.0)	<0.0001
ASTT (cm), mean (SD)*	2.4 (1.3)	2.7 (1.2)	1.8 (1.1)	<0.0001
Abdominal visceral fat (kg), mean (SD)*	1.9 (1.3)	2.2 (1.3)	1.2 (1.1)	<0.0001

*Unpaired t-test; ^†^chi-square test. SD, standard deviation; BMI, body mass index; WC, waist circumference; ASTT, abdominal subcutaneous tissue thickness.

We observed that an increase in abdominal visceral fat was associated with lower scores in the CDR-SB after adjustment for possible confounding factors (*β* = −0.79, 95% CI = −1.02; −0.57, *p* < 0.0001; Table 2). Similarly, we also found that the increase in abdominal visceral fat was associated with 54% fewer odds of cognitive impairment (OR = 0.46, CI = 0.30; 0.71, *p* < 0.0001). Sensitivity analysis confirmed that a larger amount of visceral fat was associated with lower odds of dementia (Table 3).

**Table 2:** Association between abdominal visceral fat and CDR-SB (*n* = 234).

Model	Coefficient (95% CI)	p*
I	−0.72 (−1.08; −0.36)	<0.0001
II	−0.86 (−1.25; −0.46)	<0.0001
III	−0.85 (−1.28; −0.43)	<0.0001

CDR-SB, clinical dementia rating sum of boxes; CI, confidence interval. *Negative binomial regression. Model I: adjusted for height. Model II: adjusted for height, age, sex, and education. Model III: adjusted for height, age, sex, education, diabetes mellitus, hypertension, stroke, current smoking status, current alcohol use, and physical inactivity.

**Table 3:** Odds ratio for association of abdominal visceral fat with cognitive impairment (CDR ≥0.5) and dementia (CDR ≥1).

Model	Cognitive Impairment (*n* = 234) OR (95% CI)	Dementia (*n* = 226) OR (95% CI)
I	0.44 (0.30–0.64)	0.32 (0.20–0.52)
II	0.42 (0.28–0.64)	0.30 (0.18–0.50)
III	0.46 (0.30–0.71)	0.31 (0.18–0.55)

*OR, odds ratio; 95% CI, confidence interval; p < 0.0001 for all analyses. Model I: logistic regression model adjusted for height. Model II: logistic regression model adjusted for height, age, sex and education. Model III: logistic regression model adjusted for height, age, sex, education, diabetes mellitus, hypertension, stroke, current smoking status, current alcohol use, and physical inactivity*.

**Figure 2:**
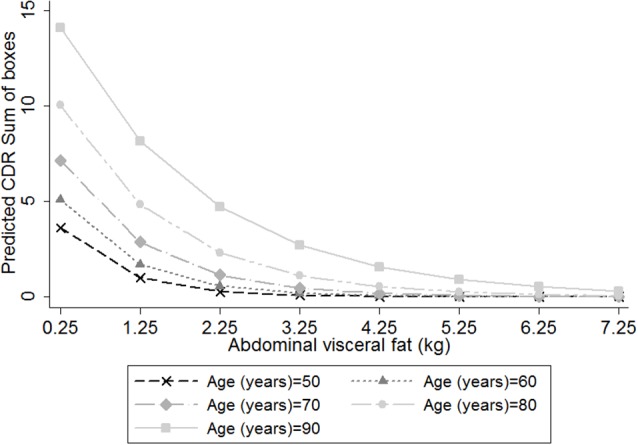
Predicted clinical dementia rating (CDR)-sum of boxes (CDR-SB) scores, according to the amount of abdominal visceral fat in individuals with different ages, considering the inclusion of an interaction term between visceral fat and age on the association between visceral fat and CDR-SB. We used negative binomial regression adjusted for height, age, sex, education, diabetes mellitus, hypertension, stroke, current smoking status, current alcohol use and physical inactivity. Age = 50 years old (cross marker); Age = 60 years old (triangle marker); Age = 70 years old (diamond marker); Age = 80 years old (circle marker); and Age = 90 years old (square marker).

**Figure 3:**
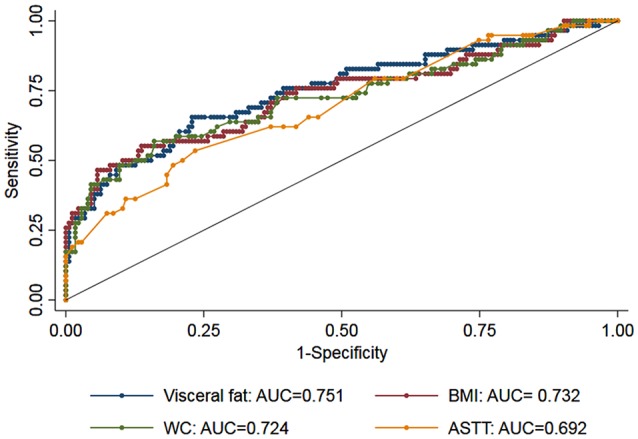
Receiver operating characteristic (ROC) curves for measurements of visceral fat (blue marker), body mass index (BMI: red marker), waist circumference (WC: green marker), and abdominal subcutaneous tissue thickness (ASTT: yellow). The area under the ROC curves (AUC) for the three measurements showed similar discrimination (*p* = 0.52).

Additionally, we observed an interaction between abdominal visceral fat and age on the association between CDR-SB and visceral fat (*β* = 0.018, 95% CI = 0.004; 0.031, *p* = 0.01; Figure 2), suggesting that smaller amounts of abdominal visceral fat were associated with higher CDR-SB scores in older individuals compared to younger ones. Regarding visceral fat accuracy to detect cognitive impairment, the best cutoff of abdominal visceral fat according to the Youden index was 1.23 with a sensitivity of 66% and specificity of 77% (Table 4). Higher visceral fat levels had also a good negative predictive value for cognitive impairment. Visceral fat (AUC = 0.754, 95% CI = 0.676–0.831), BMI (AUC = 0.729, 95% CI = 0.646–0.812), WC (AUC = 0.720, 95% CI = 0.636–0.803), and ASTT (AUC = 0.692, 95% CI = 0.612–0.773) showed good discrimination in predicting cognitive impairment with similar AUC values (*p* = 0.52; Figure 3).

**Table 4:** Accuracy of abdominal visceral fat in predicting cognitive impairment (*n* = 234).

Area under the ROC curve (95% CI)	0.751 (0.672–0.830)
Youden Index	1.23
Sensitivity	0.661
Specificity	0.771
Positive Predictive Value	0.487
Negative Predictive Value	0.865
Positive Likelihood Ratio	2.886
Negative Likelihood Ratio	0.440
Diagnostic Odds (95% CI)	6.090 (5.451–6.729)

*ROC, receiver operating characteristic; CI, confidence interval*.

## Discussion

We found that a larger amount of visceral fat was associated with lower odds of cognitive impairment and lower scores in the CDR-SB in a population-based autopsy study. Moreover, we also found an interaction between age and visceral fat on the association between visceral fat and CDR-SB scores that suggests that this inverse association was more severe in older participants. Anthropometric measurements, ASTT and autopsy-measured visceral fat showed similar discriminations for cognitive impairment.

Several studies have demonstrated that overweight or obese individuals in midlife were at a higher risk of cognitive impairment in late life (Whitmer et al., 2005; Hassing et al., 2009; Pedditizi et al., 2016). On the other hand, if adiposity and dementia were analyzed in late-life, the results are conflicting. Our results with direct measurements of abdominal visceral fat are in line with some prior studies that generally support an inverse association between obesity and cognitive impairment (Nourhashémi et al., 2003; Buchman et al., 2005; Dahl et al., 2008; West and Haan, 2009; Cronk et al., 2010; Power et al., 2011; Pedditizi et al., 2016). For example, a significant decrease in BMI (>10%) in late-life was related to a 118% greater risk of developing dementia in the following 3 years (Atti et al., 2008). Another study did not find an association between BMI and late-life dementia (Luchsinger et al., 2007), while higher BMI was related to a higher risk of cognitive impairment, especially in certain groups, as women (Gustafson et al., 2003) and those younger than 70 years (Yoon et al., 2012).

While we observed an inverse association between abdominal visceral fat and cognitive impairment using autopsy material, previous imaging studies found no association between abdominal visceral fat area and cognitive impairment (Kamogawa et al., 2010; Spauwen et al., 2017). On the other hand, a positive association between visceral adipose tissue and poor cognitive performance, but only in participants younger than 70 years (Yoon et al., 2012). Another study among 184 older adults without cognitive impairment evaluated the visceral fat and the brain structure using magnetic resonance imaging (Isaac et al., 2011). Visceral fat accumulation was associated with worse performance on memory and attention tests, and lower hippocampal volume (Isaac et al., 2011). In 1,570 older adults, visceral fat and peripheral fat mass measured by the bioelectrical impedance were related to a higher risk of severe cognitive impairment (Papachristou et al., 2015). Finally, total fat mass was evaluated using dual-energy x-ray absorptiometry, and visceral fat by computed tomography in 3,054 elderly individuals. Higher levels of adiposity were associated with worsening cognition only in men (Kanaya et al., 2009).

Although imaging studies are the most similar to ours since they measured the visceral fat, the quantification of abdominal visceral fat by autopsy is the “gold standard” because it allows the direct measurement of the visceral fat surrounding different abdominal organs (van der Kooy and Seidell, 1993). Indeed, the different findings in imaging studies could be related to the indirect measurement of visceral fat. In addition, some important differences in the sample composition need to be noted. Previous studies were performed in high-income countries (Kanaya et al., 2009; Kamogawa et al., 2010; Isaac et al., 2011; Yoon et al., 2012; Spauwen et al., 2017), while this study was performed in a low-middle income country from Latin America with great ethnic diversity and lower levels of education. Although direct measurements of visceral fat are the most accurate measurement of adiposity, we found that visceral fat, BMI, WC, and ASTT had similar discrimination to predict cognitive impairment in our sample. This finding suggests that both BMI and WC, which are accessible anthropometric measures, could be used to evaluate adiposity and the risk of cognitive impairment.

An explanation for our findings is reverse causation. A prior study showed that participants with lower BMI at baseline had lower memory scores after 10 years. Inversely, participants with lower memory scores in baseline presented a decline in BMI in the following decade. They also demonstrated that preclinical dementia may influence the body composition by weight loss even in individuals in their late 50s (Suemoto et al., 2015). Since weight loss may begin upto 20 years prior to the onset of the dementia (Knopman et al., 2007), maybe these results may be reflecting the preclinical phase of dementia. In a longitudinal study of 8-years of follow-up, lower BMI increased dementia risk in older people. However, when the dementia cases diagnosed at 1–3 years of follow-up were excluded from analysis, this relationship was not significant, suggesting that lower BMI is probably not a risk factor, but an early clinical sign of the dementia (Nourhashémi et al., 2003). In the same way, a previous autopsy study showed that the higher levels of AD neuropathology were associated with lower BMI in both participants with and without dementia. It suggests that AD pathology may contribute to weight loss even in the preclinical phase of dementia (Buchman et al., 2006). In another study, individuals with early AD had less lean mass compared with non-demented individuals (Burns et al., 2010), as well as lower weight and BMI.

Additionally, our findings of lower visceral fat with higher odds of cognitive impairment was expected since cognition and abdominal visceral fat were evaluated at the same time, and our sample was mainly of older adults. The significant interaction between age and visceral fat on the association between visceral fat and CDR-SB scores found in this study also shows that lower BMI was associated with higher cognitive impairment in older participants than in younger ones. In addition, our findings may be consequent to malnourishment associated with overt clinical dementia. Dementia may interfere with the imbalance between energy intake and energy expenditure through different mechanisms, such as damage to appetite control, forgetting to eat, refusal to eat, increased energy expenditure, apraxia, communication problems in relation to the desire of eating, impaired decision-making ability, and decreased interest in food due to apathy (Aziz et al., 2008; Droogsma et al., 2015).

Another possible explanation is that obesity is, in fact, protective against cognitive impairment due to the excess of leptin, a hormone involved in obesity pathogenesis and also related to memory and learning (Fewlass et al., 2004; Farr et al., 2006). A prior study showed that higher leptin levels were associated with greater BMI and total percent body fat. Individuals with higher levels of leptin had nearly 50% less cognitive decline compared with those with lower leptin levels (Holden et al., 2009). Additional longitudinal studies with long follow-up periods (e.g., 30 years or more) followed by autopsy, with direct measurements of visceral abdominal fat and leptin levels are necessary to clarify this association.

Based on our findings as well as on results from prior studies, we need to emphasize two points. First, we found that lower amounts of directly measured visceral fat were associated with higher odds of cognitive impairment in older participants. Therefore, it is important to evaluate carefully the cognitive function in older adults, who present with weight loss without apparent cause, because this symptom can be due to preclinical or mild dementia. Second, we found that anthropometrics measurements that are easy to obtain (BMI and WC) had similar accuracy in predicting cognitive impairment to visceral fat, which is more costly to measure since it requires an imaging exam, as computed tomography, magnetic resonance imaging or bioelectrical impedance. Thus, in settings of scarce resources as in lower middle-income countries, anthropometric measurements could be used to predict cognitive impairment. Future research should investigate the predictive value of investigating patients with weight loss for cognitive impairment early diagnosis.

Our study should be considered regarding the study limitations. This is a cross-sectional study, which limits our ability to draw causal inferences of the adiposity effect on cognitive impairment. Moreover, we did not have cognitive evaluation prior to participants’ death. To overcome this important limitation, we only included informants, who had at least weekly contact with the deceased. In addition, the evaluation with the informant was validated in clinical settings showing good accuracy for the diagnosis of cognitive impairment (Ferretti et al., 2010). In addition, information on the association between directly measured visceral fat and neuropathological lesions is not currently available. Finally, we did not have information on laboratory exams, medications, and family history. Multiple longitudinal cognitive evaluation and neuropathological information will be important to clarify the association between visceral fat and cognitive impairment in future autopsy studies. On the other hand, we should also consider the study strengths. We measured visceral fat directly in autopsy material, being possible to anatomically separate all fats and obtain the total amount of visceral fat. Additionally, our sample is from a low-middle income country with an ethnically diverse background and with low educational levels. In addition, all participants had short post-mortem intervals, which contributed to the quality of the samples. We also compared the discrimination for cognitive impairment of direct measurements of abdominal visceral with anthropometric measurements (BMI and WC) and with measurements of subcutaneous tissue obtained during the autopsy. Finally, we also excluded participants that had lost weight recently to limit the role of reverse causation. In conclusion, larger amounts of visceral fat directly measured directly in autopsy material was associated with lower odds of cognitive impairment in older adults from a lower middle-income country.

## ETHICS STATEMENT

This study was carried out in accordance with the recommendations of University of São Paulo Institutional Review Board with written informed consent from all subjects. All subjects gave written informed consent in accordance with the Declaration of Helsinki. The protocol was approved by the Comissao de Etica em Pesquisa from University of São Paulo.

## Author Contributions

AN, AC and CS conducted the data analysis and drafted the initial manuscript. AN, AC, DF-I and CS helped with results interpretation and gave critical comments for the manuscript. RN, WJ-F and CP secured funding for data collection. All authors read and approved the final manuscript.

## Conflict of Interest Statement

The authors declare that the research was conducted in the absence of any commercial or financial relationships that could be construed as a potential conflict of interest.
